# Development of a Deprescribing Intervention for Proton Pump Inhibitors in Primary Care: A Co‐Design Approach With General Practitioners and Patients

**DOI:** 10.1111/bcpt.70091

**Published:** 2025-08-19

**Authors:** Kristie Rebecca Weir, Clémentine Tombez, Yvonne Mattmann, Sofia C. Zambrano, Eliza Ferguson, Katharina Tabea Jungo, Martina Zangger, Shana Volken, Enriqueta Vallejo‐Yagüe, Renata Vidonscky Lüthold, Angela Edith Schulthess‐Lisibach, Christof Bieri, Michaela Barbier, Pascal Juillerat, Sven Streit, Jennifer Inauen

**Affiliations:** ^1^ Institute of Primary Health Care (BIHAM) University of Bern Bern Switzerland; ^2^ Sydney School of Public Health Faculty of Medicine and Health, The University of Sydney Sydney Australia; ^3^ Institute of Psychology University of Bern Bern Switzerland; ^4^ Institute of Social and Preventive Medicine (ISPM), University of Bern Bern Switzerland; ^5^ Center for Healthcare Delivery Sciences (C4HDS) and Division of Pharmacoepidemiology and Pharmacoeconomics, Department of Medicine Brigham and Women's Hospital and Harvard Medical School Boston Massachusetts USA; ^6^ Health Economics Facility, Department of Public Health University of Basel Basel Switzerland; ^7^ Institute of Pharmaceutical Medicine (ECPM), University of Basel Basel Switzerland; ^8^ Gastroenterology, Clinic for Visceral Surgery and Medicine, Inselspital, Bern University Hospital University of Bern Bern Switzerland; ^9^ Crohn and Colitis Centre, Gasteroenterology Intesto Bern and Fribourg Switzerland

**Keywords:** behavioural intervention, communication, deprescription, medication, theoretical domains framework

## Abstract

Proton Pump Inhibitors (PPIs) are widely prescribed medications globally. PPIs are often inappropriately used – for example, prescribed without a clear indication, at higher‐than‐necessary doses, or for longer durations than needed – resulting in increased risks of adverse health outcomes such as nutrient deficiencies, osteoporosis‐related fractures, and kidney disease. The effectiveness of current deprescribing interventions is inconsistent; this may relate to the misalignment with behavioural mechanisms, supporting the need for a mechanism‐driven approach that targets key behavioural determinants. We co‐designed a PPI deprescribing intervention with patients and health professionals for the Swiss primary care setting. This involved identifying behavioural determinants of PPI deprescribing from the literature at both the general practitioner (GP) and patient levels, mapping them to behaviour change techniques, selecting intervention elements, and developing contextualised intervention tools to effectively influence these determinants. The intervention tools were iteratively developed and assessed through qualitative methods with 16 patients and 18 health professionals. Participants considered the tools to be acceptable and practical for use in the Swiss primary care context.

## Summary

Proton Pump Inhibitors (PPIs) are commonly prescribed, in Switzerland and worldwide, beyond the recommended short‐term use, which comes with significant health risks for patients. Current deprescribing efforts may be less effective because they do not fully address key factors influencing general practitioners' (GPs) and patients' medication decisions. To address this, we developed a deprescribing intervention tailored to the context of Swiss primary care. Our approach involved identifying enablers and barriers to deprescribing PPIs, selecting intervention strategies to address those enablers and barriers and creating tools to support safer, more effective PPI use.

## 1 Background

Proton Pump Inhibitors (PPIs) are among the most frequently prescribed medications worldwide [1, 2]. PPI use in Switzerland is increasing, with almost one quarter of adults receiving a PPI prescription in 2017 [3]. Prolonged use of PPIs is often unnecessary and reducing or discontinuing PPIs is typically recommended after four to eight weeks [4]. Long‐term use of PPIs has been associated with a range of adverse health outcomes, including nutrient deficiencies (such as vitamin B12, vitamin D and magnesium), increased risk of osteoporosis leading to bone fractures, Clostridioides difficile and other intestinal infections and pneumonia [5, 6, 7, 8, 9, 10]. The high rates of inappropriate use, combined with the potential for harm, emphasise the need for deprescribing – the process of reducing or discontinuing PPIs that are no longer indicated.

PPI deprescribing interventions are inconsistently effective, with significant variability in outcomes across studies. A systematic review of 21 studies identified only six effective interventions for reducing PPI use, while the rest showed inconclusive results or no benefit [11]. Another systematic review reported deprescribing success rates ranging from 14% to 64% [12], highlighting the challenge of identifying consistently effective strategies. Variability in study design, settings and intervention components prevents meaningful cross‐study comparisons and limits definitive conclusions about optimal approaches to PPI deprescribing.

One possible reason for the inconsistent effectiveness of deprescribing interventions is the limited consideration of the underlying behavioural determinants that influence deprescribing. Ailabouni and colleagues [13] identified a gap in the literature: most deprescribing interventions do not fully explore or explain the determinants driving their outcomes. Applying behavioural theory during intervention development can help identify key determinants, understand mechanisms of change and address implementation barriers.

Two systematic reviews retrospectively examined the role of behaviour change techniques (BCTs) in deprescribing interventions [14, 15]. The first review [15] analysed 25 studies, finding that deprescribing interventions had variable effects on reducing medication use and inappropriate prescribing, with goal setting, social support, knowledge shaping and behavioural comparisons linked to greater effectiveness. The second review [14] mapped 43 primary care deprescribing studies to the Behaviour Change Wheel, identifying a wide range of BCTs, with environmental restructuring, enablement and persuasion being the most commonly used. Both reviews emphasised the need for further research to identify which BCTs are most effective in supporting deprescribing in primary care, noting the limitations of applying theory to the interventions in a retrospective manner. Several theory‐driven interventional studies are currently underway in the United Kingdom (UK), using robust theoretical frameworks and evidence to target appropriate medication use in older adults, namely adherence [16, 17, 18], appropriate prescribing [19, 20, 21] and deprescribing [22, 23, 24, 25].

Beyond identifying behaviour change determinants and mapping them to BCTs (mechanism‐driven), effective interventions are also tailored to the persons that will be using the intervention, e.g., by using the Person‐Based Approach [26]. This approach is based on in‐depth understanding and personalising an intervention to the needs of the target population using qualitative research. Accordingly, we applied a systematic, mechanism‐driven and person‐based approach to develop a behaviour change intervention for the DROPIT Trial (‘Deprescribing Inappropriate Proton Pump Inhibitors Trial’). This unblinded, cluster‐randomised controlled trial (RCT) is currently being conducted in primary care settings across the German‐speaking regions of Switzerland (trial details published elsewhere [27]). This intervention was designed to guide GPs and patients through the process of deprescribing inappropriate PPIs. We aimed to understand the behaviours and their determinants related to PPI deprescribing at the GP and patient level and map these to BCTs (Stage 1), select intervention elements (Stage 2), and develop intervention tools (Stage 3) that are contextualised to the needs of GPs and patients, with the potential to change the behavioural determinants.

## 2 Materials and Methods

This study was reviewed and approved by the Ethics Committee of the Faculty of Human Sciences of the University of Bern [2022‐09‐01]. The study was conducted in accordance with the Basic and Clinical Pharmacology and Toxicology policy for experimental and clinical studies [28].

### 2.1 Study Design

**FIGURE 1:**
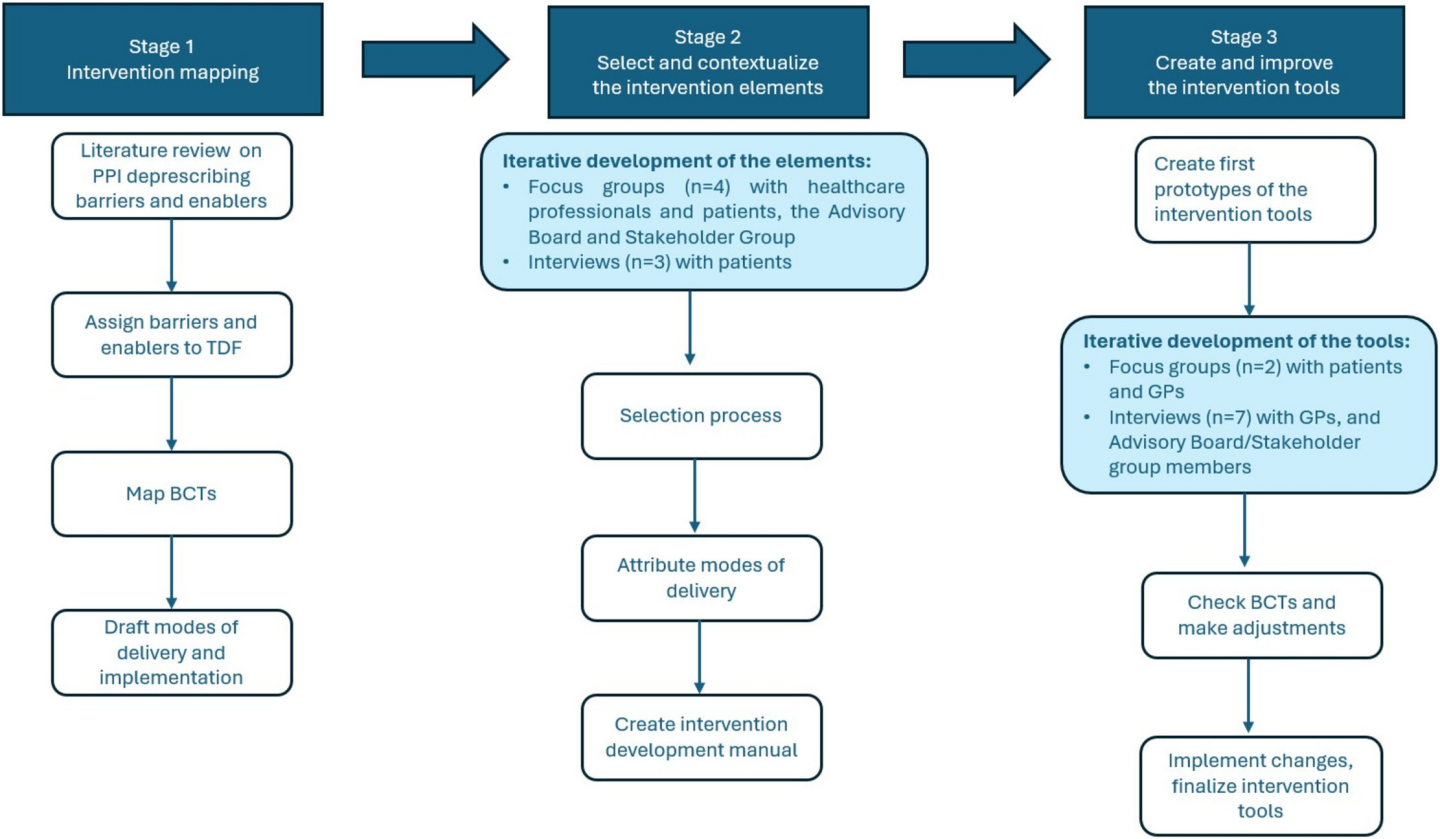
Flow chart DROPIT intervention development. PPI = Proton Pump Inhibitors; TDF = Theoretical Domains Framework [29]; BCTs = Behaviour Change Techniques [30, 31].

Based on the Person‐Based Approach [26], we adopted a research approach that considered the perspectives of the target population, GPs and patients, to develop the DROPIT intervention. This involved integrating in‐depth qualitative research with iterative feedback from stakeholders, including patients, consumer group representatives, GPs, pharmacists, gastroenterologists and researchers in public health, epidemiology, psychology, health communication, clinical research and health economics, through a participatory co‐design process. The development process was divided into three distinct stages, summarised in Figure 1.

### 2.2 Project Team

A multidisciplinary team guided the intervention development. The team included experts in psychology (JI, SCZ, CT, YM, EF and SV), primary care (SS, MZ and CB), gastroenterology (PJ), pharmacy (AESL and EVY), public health (KRW, KTJ and RVL), nutrition (RVL), health communication (KRW and SCZ), pharmacoepidemiology (KTJ, EVY and AESL) and health economics (MB). The DROPIT intervention core team consisted of researchers responsible for the intervention development.

The DROPIT Trial [27] is supported by an active Advisory Board and Stakeholder Group consisting of patients, clinicians and researchers from seven countries: Switzerland, France, Netherlands, Italy, Belgium, Canada and the USA. Regular meetings and feedback sessions were held to inform the study design, guide the interpretation of findings and to shape the development of the intervention, ensuring alignment with stakeholders' needs and priorities.

### 2.3 Participants

Participants were patients with experience with PPIs and health professionals. The Advisory Board and Stakeholder participants (presented together hereafter) had expertise in primary care, internal medicine, gastroenterology, pharmacy, public health and were patient representatives. Health professionals and patient participants were recruited via multiple avenues, including professional networks, social media platforms and public website advertisements and they were reimbursed for their participation.

Box 1 summarises the methods used and participant groups from Stages 2 and 3. Stage 1 involved a review of the literature, so no participants were included at this stage.

**Box 1:** Overview of study methods and participants.

Method	Stage 2	Stage 3	Total
Focus groups	4	2	6
Interviews	3	7	10
Participants	Patients (*n* = 7) Health professionals ^a^ (*n* = 4) advisory and stakeholder participants (*n* = 10)^b^ *11 women*, *10 men*	Patients (*n* = 7) GPs (*n* = 6) advisory and stakeholder participants (*n* = 2)^b^ *9 women*, *6 men*	36 *20 women*, *16 men*

^a^ Health professionals (*n* = 4) with backgrounds in general practice, pharmacy, and gastroenterology.

^b^ Advisory and Stakeholder participants (*n* = 12) had expertise in primary care, internal medicine, gastroenterology, pharmacy, public health and patient representatives.

### 2.4 Intervention Development Process

#### 2.4.1 Stage 1: Intervention Mapping

To identify behavioural determinants of PPI deprescribing at both patient and GP levels, we conducted a targeted literature review using PubMed and reference lists of key papers to identify relevant studies on barriers, enablers and existing interventions. We extracted information on study design, intervention strategies and reported effectiveness. This data was organised in a comprehensive document. We drafted the target behaviours at GP and patient level considered necessary to perform deprescribing, based on the project team's expertise. Two behaviour change experts then wrote definitions of the barriers and enablers based on the respective studies that described them. Using these definitions, the experts coded the barriers and enablers according to their domains, based on the Theoretical Domains Framework (TDF) [29]. This coding exercise facilitated the understanding of the behavioural determinants and their mapping to relevant intervention functions and BCTs using the Behaviour Change Wheel [32]. Finally, concrete implementation strategies were drafted for the identified behavioural determinants and BCTs, also accounting for the effective intervention elements identified in the review of the intervention literature.

#### 2.4.2 Stage 2: Selection Process of Intervention Elements

In Stage 2, interviews and focus groups were conducted to identify the most promising intervention elements and to gather insights into the validity of the behavioural determinants of PPI deprescribing identified in Stage 1. Further, participants' ideas for additional intervention elements were considered. The focus groups, which included a mix of patients and health professionals, began with a vignette illustrating a potential case of PPI deprescribing, followed by discussion guided by the presentation of intervention elements from Stage 1.

The data was analysed using directed qualitative content analysis [33]. The intervention elements formed the basis of the coding framework and the data were coded according to their relevance to each element, identifying supportive or unsupportive statements, delivery methods and potential barriers. The data was then summarised for each intervention element. This analysis approach helped to identify intervention elements suitable for inclusion in the trial in terms of their acceptability and perceived effectiveness and key considerations for their implementation. The most promising intervention elements identified through the qualitative content analysis were reviewed by the intervention core group, focusing on feasibility for the trial and assigning modes of delivery. This information formed the intervention manual, a detailed document that guided prototype development for the intervention tools.

#### 2.4.3 Stage 3: Development of the Intervention Tools

In Stage 3, initial prototypes of the intervention tools were developed by the study team, with active involvement from patients, GPs, Advisory Board and Stakeholder Group participants. Overall, input gathered through focus groups, interviews and email feedback informed both the content (e.g., messaging, tone) and presentation (e.g., visual layout) through iterative refinement. A visual designer developed the layout and visual style of the intervention tools. The digital symptom diary was implemented with support from a software engineer and research assistants and the teaching videos were produced with a multimedia specialist.

Participants were asked to envision themselves using the intervention elements in practice. The feedback focused on the usability, feasibility and practical application of the intervention elements, with particular emphasis on ensuring that all patient‐facing information was clear and understandable. Patients reviewed materials designed for them (brochure and screenshots of the digital symptom diary), while GPs and health professionals from the Advisory Board and Stakeholder Group reviewed the content intended for GPs (PPI deprescribing algorithm (decision tree), infographic/script for the consultation, teaching module including symptom diary for patients and training session).

Interim analyses were conducted after the Stage 3 focus groups and interviews to identify necessary changes to the intervention prototypes, leading to iterative modifications. Focus group analysis was informed by the interviewers' immediate reflections and notes, while interviews were analysed using the Framework Method [34]. We subsequently conducted a thematic analysis, drawing on Braun and Clarke's six‐stage approach to guide nuanced modifications: familiarisation with the data, generating initial codes, searching for themes, reviewing themes, defining and naming themes and presenting the findings [35].

If necessary, the intervention tools were (re‐)coded in parallel with the iterative adaptation using the BCTs Taxonomy [7] (BCT Check). This included coding new or adapted intervention elements to ensure the accuracy of the intervention description, with changes documented (Data S1).

## 3 Results

### 3.1 Stage 1

We identified empirical studies examining patients' and GPs' perspectives on barriers and enablers of deprescribing [36, 37, 38, 39]. We included unpublished results from Swiss qualitative studies by master students [40, 41] on the perceived barriers and enablers of PPI use from the perspectives of patients (*n* = 7), GPs, and pharmacists (*n* = 12), supervised by two co‐authors (KTJ, SS). Barriers and enablers were categorised as patient‐ or GP‐level factors and summarised in a table with definitions (see Data S1). A total of 33 factors were identified. Examples of barriers included GPs' fear of negative reactions from the patient and lack of opportunities to review medications. Examples of enablers included confidence in managing recurring symptoms and feeling comfortable questioning a specialist's PPI prescription.

During TDF coding, five of the 33 identified barriers/enablers were split into separate factors to better distinguish their psychological aspects and intervention approaches (see Data S1). One barrier related solely to prescribing and was therefore excluded. This process resulted in 40 barriers and enablers; 14 were at the patient level and 26 were at the GP level. They referred to a diverse range of behavioural determinants: knowledge, skills, beliefs about consequences and capabilities, reinforcement, goals, intentions, memory, attention and decision processes, environmental context and resources, social influences, social/professional role and identity, emotions and behavioural regulation. The behavioural determinants were then mapped to intervention functions and BCTs. Finally, implementation strategies and modes of delivery were proposed, including a PPI deprescribing consultation, a PPI deprescribing algorithm (decision tree), a consultation script, a teaching module and training materials, a behavioural contract, a brochure, a flyer, educational videos, a prompting system and a symptom diary (see Data S1).

### 3.2 Stage 2

In Stage 2, we identified key perspectives from 21 participants (see Box 1) on proposed intervention elements and mode of delivery, as well as their ideas for additional elements.

Patients agreed with the importance of receiving transparent information about deprescribing, including the rationale, potential negative side effects, and the possibility of rebound symptoms: *‘* *I want to know the advantages and disadvantages, what we'll do if the problem reoccurs, what options I have, and what I should expect*’ (Patient, ID1, Focus Group). They also agreed with the value of having opportunities to discuss and co‐create deprescribing plans with their GP, seeking peer support from other patients and having information on alternative treatments and their implementation: ‘*the recommendations for alternative strategies…This is the most important point for me*’ (Patient ID1, Focus Group). Additionally, some patients endorsed having a tool for monitoring their symptoms and to support communication with their GP. GPs agreed with the importance of an intervention that fostered empowerment and shared decision‐making.

GPs also saw the importance of external motivation to encourage the use of visual aids, such as factsheets and graphics on PPI deprescribing in daily practice. They appreciated the relevance of up‐to‐date, scientific evidence to underpin deprescribing decisions. Input from specialists was considered most useful when it was evidence‐based and positively framed, focusing on potential benefits for patients rather than risks. Peer testimonials were also seen as valuable for building confidence and motivation. However, implementing digital symptom monitoring for patients was perceived as potentially challenging: ‘*It makes sense, theoretically, to have [a digital symptom diary] …. As a clinician, I am very interested in having indicators for my practice, but it should be integrated into my clinical process and ideally should not require additional work*’ (GP, ID1, Focus Group). GPs noted that feedback on deprescribing outcomes can be a strong motivator for engaging in deprescribing efforts, while patient feedback on the process is important for effective communication and providing appropriate support.

Within this stage, several intervention elements had been discussed and received support from stakeholders were omitted for the following reasons:

- Peer support for patients: Suggested forums, group discussions and self‐help groups were omitted due to anticipated implementation challenges and ethical concerns about unmoderated medical advice.
- Follow‐up appointments: Regular follow‐up appointments were omitted as part of the intervention due to practicality issues for GPs and cost considerations. Instead, GPs would be encouraged to offer follow‐up appointments at their discretion, especially for complex cases.
- Audit and feedback for GPs: Audit and direct feedback systems to prompt GPs to review PPI prescribing was omitted due to implementation difficulties with diverse practice information systems. Instead, GPs would be encouraged to set up their own reminder systems.
- Involving practice staff in deprescribing: Delegating deprescribing tasks to other practice staff was omitted due to variability in training and because it was considered to be outside the scope of this study. However, GPs could involve staff for simpler cases while handling more complex cases themselves.

Additionally, the following behavioural determinants were not included in the intervention:

- Intentions: Patients' lack of intention or unwillingness to try suitable alternatives to PPIs was identified as a barrier to deprescribing. While strategies like behavioural contracts were considered to support commitment, they were ultimately omitted, as GPs reported they would not use them in practice.
- Social/Professional Role and Identity: Encouraging GPs to reflect on their deprescribing practices and reframe their identity through peer comparisons and self‐reflection was deemed infeasible due to the individual nature and time constraints in clinical practice.

With these elements and behavioural determinants removed, Stage 3 continued with the development of the remaining ones (Data S2).

### 3.3 Stage 3

**FIGURE 2:**
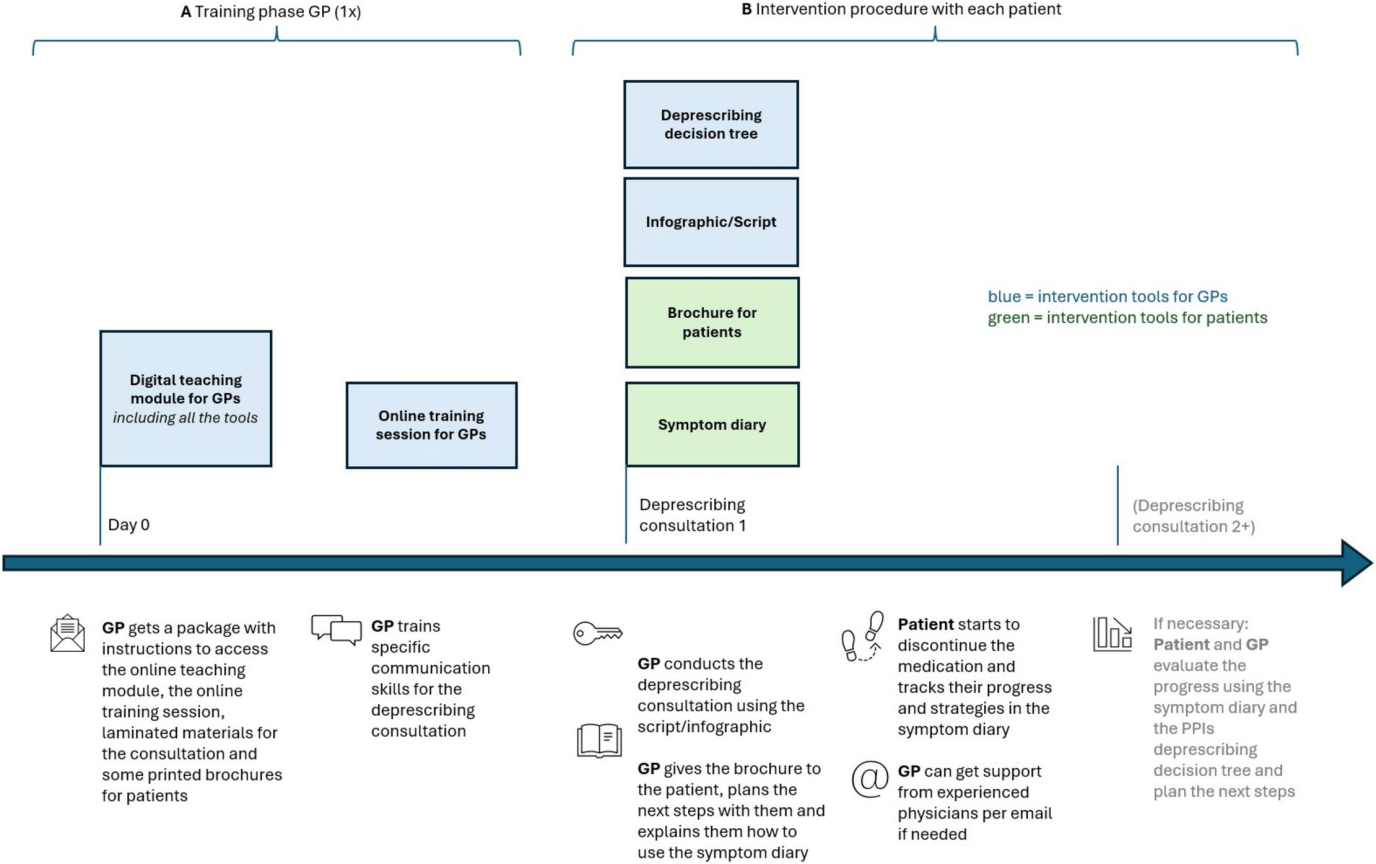
Planned delivery of the study intervention. A. Training phase for each GP included a teaching module and a training session. B. Intervention procedure delivered individually by the GP with each patient.

Initial prototypes of the intervention within specific delivery modes were developed based on the Stage 2 findings (Figure 2). For patients, this included a brochure and digital symptom diary, while for GPs, a decision tree, infographic, script, online teaching module and an online training session were developed. The original prototype included a flyer for patients highlighting potential side effects of PPIs; however, it was removed based on logistical considerations and patient feedback indicating it could cause unnecessary worry.

The results of the interim analysis that guided iterative adaptations of the intervention tools will be presented; followed by the results of the subsequent thematic analysis.

In the interim analysis, three key areas for improvement of the intervention tools were identified:

1. ***Simplify and improve practicality***: Both patient‐facing and GP‐facing materials needed simplification. For patients, this meant revising the brochure with simpler terminology, improved layout and larger font sizes. Patients recommended offering both digital (app) and paper‐based symptom diary options to accommodate varying preferences and capabilities. GPs found the teaching module impractical for routine use, as it was too detailed and complex for standard consultations. Additionally, all participants reported that the section on planning deprescribing consultations was not useful, as GPs rarely had the opportunity to plan for individual sessions in advance.
2. ***Empower GPs***: Some GPs felt the tone was condescending in sections of the teaching module, undervaluing their expertise. Participants felt the content could overemphasise potential barriers or shortcomings in deprescribing conversations, rather than reinforcing GPs' existing skills and expertise in this area.
3. ***Strengthen emphasis on shared decision‐making***: Patients reported wanting to feel supported by their GPs during the deprescribing process (e.g., for the GP to listen, answer questions, and provide explanations about the possibility of rebound symptoms). Additionally, several health professional participants believed the teaching module did not sufficiently highlight patient autonomy in PPI deprescribing decisions. They felt the materials should place greater emphasis on shared decision‐making between GPs and patients, rather than positioning deprescribing as a directive process.

Based on this interim analysis, modifications were made to improve the deprescribing intervention. A two‐sided infographic and script were developed to facilitate use during consultations, and the graphic designer addressed layout and formating issues. The teaching module and online training were shortened and simplified. The teaching module was revised to use more empowering, non‐lecturing language, while sections on GP‐related barriers were refocused as strategies that support deprescribing. Content was also reformulated to reduce pressure on both GPs and patients to achieve successful deprescribing, and a stronger emphasis on shared decision‐making was incorporated.

#### 3.3.1 Thematic Analysis

**FIGURE 3:**
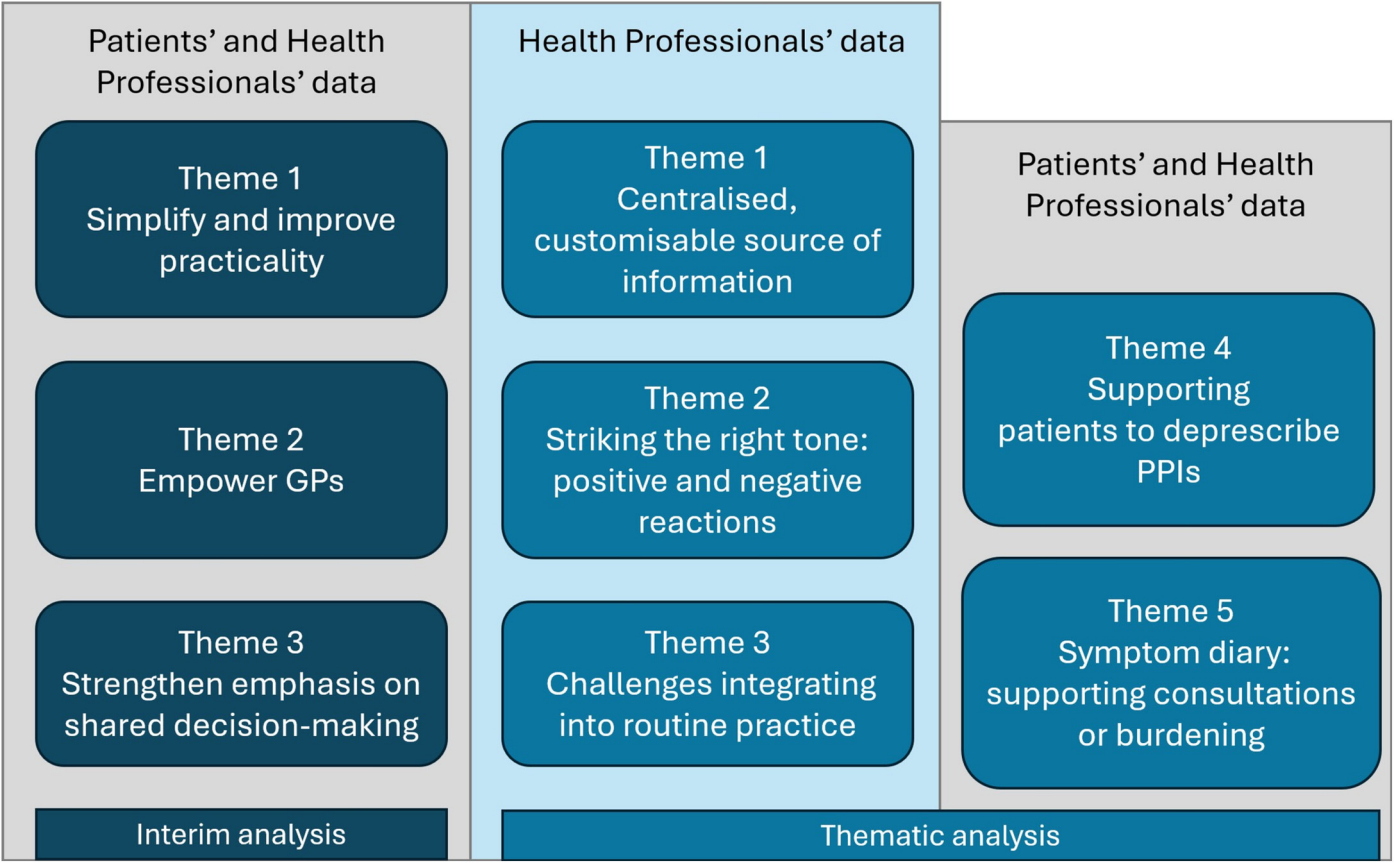
Themes reflecting participants' perceptions of the intervention tools and their sources. ‘Health professionals’ were comprised of GPs and health professionals from the Advisory board and Stakeholder group.

From the thematic analysis, we developed five key themes reflecting participants' perceptions of the intervention tools (Figure 3). This analysis confirmed the interim findings and provided new insights, allowing further refinement of the intervention.

##### 3.3.1.1 Theme 1: Centralised, Customisable Source of Information

The GPs and health professionals from the Advisory Board and Stakeholder Group viewed the intervention tools as a comprehensive and consolidated source of information on PPI deprescribing. Even after adjustments to shorten it, some health professionals felt the intervention tools, particularly the GP teaching module, contained too much detail, while others valued its thoroughness. For example, one GP shared the following perspective on the teaching module: ‘…*when I first read it, I thought, whoa, this is really quite detailed, right? But then, as I read through it, I also thought, yes, it's actually very good, what's written there, yes, very interesting*’ (GP, ID2 Interview).

Additionally, many GPs reflected that they could extract the information which was most relevant or novel to them: ‘…*there are always individual things that are perhaps newer or that were no longer on the radar…if you really want to give patients better advice, then it's certainly good if you have taken a more serious look at it again*’ (GP, ID3, Focus Group).

The teaching module's flexibility was widely appreciated by GPs and members of the Advisory Board and Stakeholder Group, as it could allow clinicians to focus on the sections most relevant to their needs and discussions with patients: ‘*Well, I feel that it is quite user‐friendly. What I really like is that I can decide for myself what I want to do and what not and if there are things I do not need*’ (GP, ID4, Focus Group).

##### 3.3.1.2 Theme 2: Striking the Right Tone – Positive and Negative Reactions

This theme explored the varied responses that GPs had to the tone and approach of the intervention tools, highlighting both positive and negative responses. Two sub‐themes captured these perspectives.

###### 3.3.1.2.1 Empowered and Motivated

Some GPs liked the interventions' practical guidance on deprescribing, making it *“clear…how [GPs] can tackle the problem”* (GP, ID4, Interview) and helping them feel empowered and motivated to address the issue. Similarly, an Advisory‐Stakeholder member emphasised how the intervention addresses the complexities of deprescribing in a way that *“lowers the barriers for GPs, because they somehow get the feeling, ‘ah yes, exactly, people know what problems I have’”* (Advisory Board and Stakeholder Group member, ID1, Interview).

###### 3.3.1.2.2 A Call for Considerate and Individualised Approaches

In contrast, other GPs and Advisory‐Stakeholder members experienced aspects of the intervention as potentially demotivating or condescending. They emphasised the importance of a more individualised approach that recognises the challenges of deprescribing without oversimplifying them. One participant commented, ‘*This is not a* *competitive sport…it's kind of great if you even try. And it is also okay if you fail*’ (Advisory Board and Stakeholder Group member, ID1, Interview).

A couple of participants criticised the tone of specific sections. One statement in particular was identified as condescending: ‘*Even the most experienced GPs do not always succeed in taking individual factors into account*’, about which one GP remarked: ‘*Yes, that is our daily bread…I think this sentence is nonsense…everyone knows that, you do not have to have it in writing*’ (GP, ID5, Interview).

##### 3.3.1.3 Theme 3: Challenges Integrating Into Routine Practice

GPs and Advisory‐Stakeholder members had concerns about the practicality of incorporating the intervention tools into routine consultations. Many GPs found the recommendations for planning and structuring conversations to be unrealistic, given the dynamic nature of patient interactions. As one GP explained: ‘…*it's kind of a dynamic thing…that does not help me either, if I have such a flowchart, if I fall out of it, then…I could not use that as a help*’ (GP, ID6, Interview). Additionally, participants highlighted that deprescribing PPIs is often just one of many issues addressed during a typical consultation, making it difficult to allocate time for structured deprescribing discussions.

##### 3.3.1.4 Theme 4: Supporting Patients to Deprescribe PPIs

This theme identified how patients can be supported during the PPI deprescribing process, drawing from the perspectives of GPs, members from the Advisory board and Stakeholder Group and patients themselves.

An Advisory‐Stakeholder member suggested that the GP's training module should place greater emphasis on addressing patient needs rather than focusing mainly on the GP's goal of deprescribing. They felt the original prototype did not go far enough in representing patient perspectives and emphasised the importance of shared decision‐making.

Some GPs questioned the necessity of specific intervention elements, such as communication guides, expressing confidence in their ability to support their patients in the process of PPI deprescribing without such scaffolding. One GP explained: ‘…*when I explain to the patient…in my own words why we are taking the whole thing [the PPI]…then he has so much trust in me that he simply does it (deprescribe) without me having to explain to him much with some decision trees…he just does it and they say if the doctor says “stop”, then it will be like that*’ (GP, ID7, Interview).

Patients expressed a strong need for information and support regarding PPIs. They appreciated the alternative strategies outlined in the patient brochure. However, some voiced concerns about feeling unsupported during the deprescribing process. One patient, for example, expressed apprehension about being left to manage on their own after the intervention: ‘…*with the alternative things [strategies], it sounds to me like I am being informed and now “you see for yourself somehow so that you can cope with it”…. If I got that, I'd think, “it's just me again*”’ (Patient, ID2 Focus Group).

In contrast, another patient noted that the inclusion of testimonials in the materials helped alleviate feelings of isolation, which they found valuable.

##### 3.3.1.5 Theme 5: The Symptom Diary: Supporting Consultations or Burdening?

The symptom diary, available in both digital (app) and paper formats, was designed for patients to use. The GPs' training includeds instructional videos and a training session on how to introduce and explain the diary to patients. The symptom diary was viewed by several patients and Advisory‐Stakeholder members as a valuable tool for fostering collaboration and strengthening the doctor‐patient relationship. They said that using the diary could improve preparedness for consultations and save time for both GPs and patients. This was illustrated by one patient, who remarked: ‘*I have the feeling that if you answer the diary… you somehow go into the doctor's consultation in a completely different way. Clearer, and it also saves time for both sides*’ (Patient, ID3 Focus Group). However, some GPs expressed reservations, highlighting the potential administrative burden associated with the diary. They suggested that direct discussions about symptoms might be more practical and less time‐consuming.

Opinions on the likely degree of adherence to the symptom diary varied. One patient felt the diary's concise format and digital option would encourage its use: ‘*it is kept so short, the willingness is already high. Because it's relatively a short thing*’ (Patient, ID4, Focus Group). Conversely, others suspected that some patients may not use the diary, regardless of its format. Three GPs, reflecting on prior experiences with similar tools, anticipated that patient engagement with the symptom diary would be limited.

### 3.4 Description of Finalised Study Intervention

**FIGURE 4:**
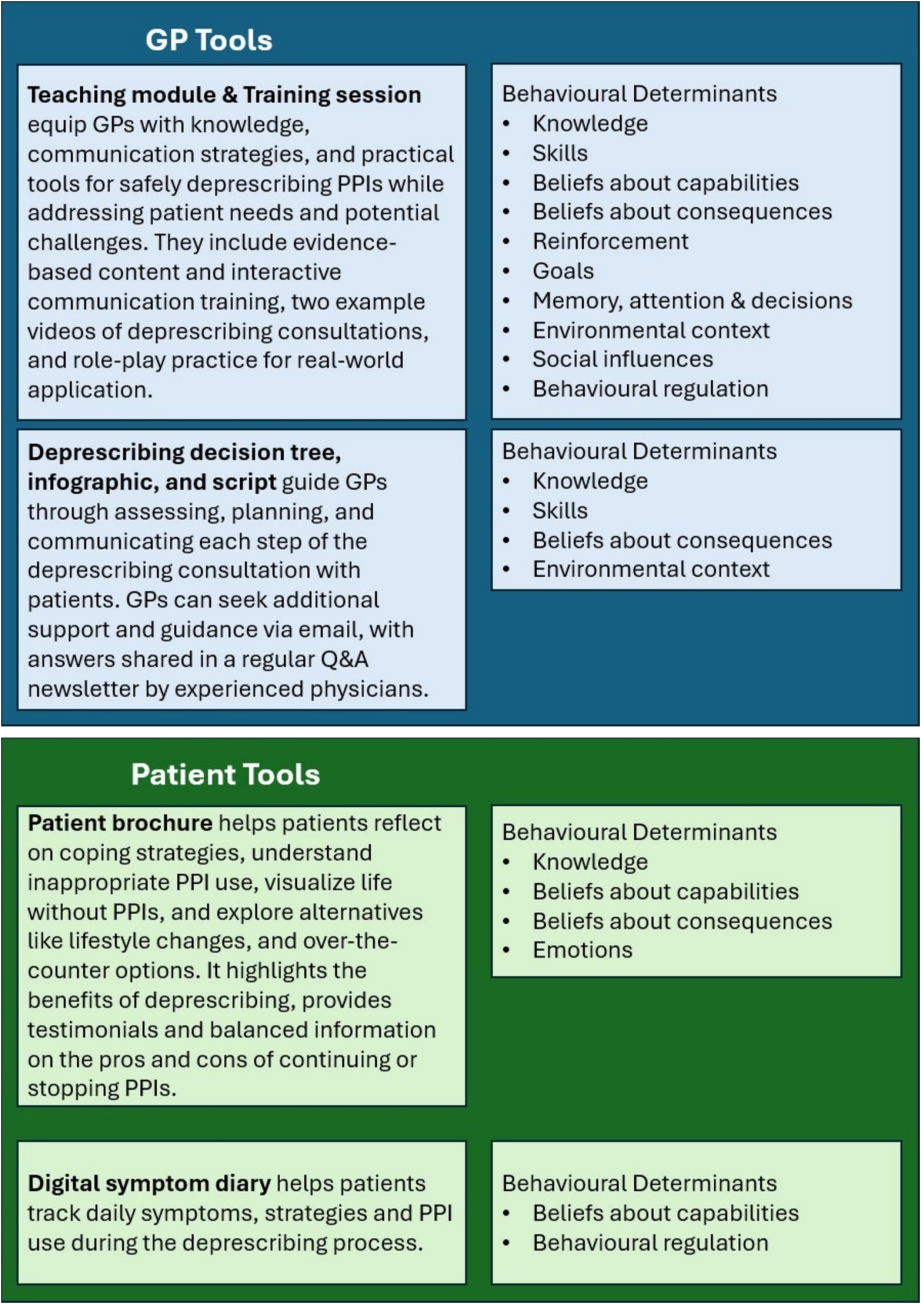
(a) Description of the intervention tools and their behavioural determinants. ‘Health professionals’ were comprised of GPs and health professionals from the Advisory board and Stakeholder group.

Overall, the intervention included a training phase for each GP, followed by an intervention phase delivered individually with each patient. The intervention tools (Figure 4a,b) for GPs included a teaching module (both digital and printed versions) and an online training session on PPI deprescribing. The teaching module contained two teaching videos of PPI deprescribing consultations between a GP and a patient. GPs would also receive an infographic and a conversational script, a deprescribing decision tree and a printed patient brochure for use during deprescribing consultations. They may also receive additional support and guidance via email, with answers shared in a regular Q&A newsletter by experienced physicians. The patient brochure contained information about PPIs and deprescribing, as well as a QR code directing patients to a digital app for self‐monitoring symptoms using the digital symptom diary, with a paper‐version available. See Data S2 for a complete description of the intervention tools, their associated behavioural determinants and BCTs with specific implementation strategies. The intervention tools are in German.

## 4 Discussion

We developed a behaviour change intervention to initiate PPI deprescribing in the context of the DROPIT trial, contextualised to the Swiss primary care setting using qualitative methods. Through a mechanism‐ and person‐based approach, we identified key behavioural determinants and co‐designed the intervention tools with stakeholders. Finally, we assessed the acceptability and feasibility of the intervention. The comprehensive intervention targets key behavioural determinants and addresses them using BCTs integrated into multiple intervention tools. These tools include training for GPs, a decision tree for identifying the medical indication for deprescribing, resources to support the deprescribing discussion with patients (brochure, infographic, script) and a symptom diary for patients to monitor recurring symptoms.

The intervention aims to target a broad range of deprescribing determinants for GPs and patients that spans the behaviour change process: determinants for building intentions to deprescribe (e.g., knowledge, beliefs about consequences and abilities and emotions), making the deprescribing decision (goals) and implementing and maintaining deprescribing behaviour (e.g., behavioural regulation and memory processes). It also targets supportive contextual influences (environmental context and including social support), which are important for successful behaviour change [42]. The DROPIT trial [27] will test the hypothesis that the intervention's comprehensiveness will lead to high efficacy in deprescribing inappropriate PPIs.

To our knowledge, this is among the first PPI deprescribing intervention targeting GPs and the general patient population to be developed in Swiss primary care using a theory‐ and evidence‐based co‐design approach. It is encouraging that similar theory‐driven approaches are being used to develop interventions for comparable contexts. In the UK, for example, there are four studies targeting medication use in older adults, focusing on adherence [16, 17, 18], appropriate prescribing [19, 20, 21] and deprescribing in the clinical setting [22, 23, 24]. Scott and colleagues' [22, 23, 24] hospital‐based deprescribing intervention targets geriatricians and pharmacists and focuses on systemic and provider‐driven deprescribing strategies. Cadogan and colleagues' [19, 20, 21] GP‐focused intervention for appropriate prescribing focuses on education and decision support, without direct patient engagement. Patton and colleagues' [16, 17, 18] pharmacy‐delivered intervention for patients emphasises self‐monitoring and patient engagement through tools like a paper‐based symptom diary. Radcliffe and colleagues' [25] deprescribing intervention targets older patients with frailty and integrates clinician, patient and carer components. It includes a deprescribing tool and identification system to support clinicians in selecting patients and prioritising medicines, a pre‐consultation leaflet to engage patients and carers and a structured medication review with tailored follow‐up and monitoring. These studies have also arrived at broad sets of behavioural determinants to target in their interventions and RCTs to assess their effectiveness are either underway or planned. Taken together, findings from these studies and the DROPIT trial (once completed) will provide valuable insights into how complex interventions can effectively promote appropriate prescribing and deprescribing by targeting key behavioural determinants.

### 4.1 Reflections on the Intervention Development Approach

A strength of the theory‐ and evidence‐based co‐design approach is that the intervention integrates health psychology principles and qualitative research representing the perspectives of the persons involved in deprescribing. A co‐design process with patients and health professionals enabled iterative refinement of the tools, with input from a multidisciplinary, international team. Meaning the intervention is designed to address behaviour change mechanisms of deprescribing in the target population, based on the available knowledge.

Developing a multifaceted intervention tailored to the needs of both patients' and GPs' requires substantial time and resources, which diverts resources and limits the ability to conduct other foundational steps, such as randomised pilot or implementation studies. Our approach prioritised stakeholder engagement and co‐design over early prototype testing, meaning the intervention will undergo its first real‐world evaluation in the DROPIT trial. Small‐scale prototype testing could have been conducted as an additional step during or after intervention development to identify usability issues and refine the intervention ahead of a full‐scale trial [27], but was not feasible within the available time and resource constraints. While we assessed the acceptability of the developed intervention tools to some extent, their use in clinical practice remains untested and challenges with recruitment, delivery or uptake may be encountered during the trial or implementation phases. Future research should explore whether the added investment in such a resource‐intensive intervention development has greater efficacy than more streamlined approaches to determine whether the additional effort is justified.

While considering the diverse perspectives of GPs and patients when designing the intervention is a strength, this comes with the challenge that needs and perceptions of GPs and patients can vary across and within these groups. In our study, GPs expressed differing views on the level of detail and structure provided by the intervention tools – some appreciated their thoroughness, while others found them overly detailed or prescriptive. Patient perspectives on the intervention tools also varied, with some finding them empowering and helpful, particularly the testimonials, whereas others felt insufficiently supported by the same resources. These differences suggest that a flexible intervention is needed to accommodate the diverse preferences, experiences and consultation styles of GPs, as well as the varying needs of patients. We did not formally assess health literacy levels of the patient‐facing materials, so their accessibility for individuals with lower health literacy is unknown. In addition, our literature review was not a formal systematic review and some relevant studies that could have informed the intervention development may have been missed.

The DROPIT intervention was developed using a theory‐ and evidence‐informed approach, integrating feedback from a multidisciplinary team and key stakeholders to address inappropriate PPI use in Swiss primary care. The intervention provides GPs with a teaching module, training session, infographic, conversational script and deprescribing decision tree, while patients receive a brochure and digital symptom diary. The intervention tools were iteratively refined and evaluated with patients and health professionals, who perceived them to be acceptable and feasible for use in primary care. Further testing will assess the effectiveness and impact of the intervention on deprescribing practices through the DROPIT trial, a cluster‐randomised study currently underway.

## Author Contributions

Kristie Rebecca Weir, Clémentine Tombez, Yvonne Mattmann, Sofia C Zambrano, Eliza Ferguson, Katharina Tabea Jungo, Martina Zangger, Shana Volken, Enriqueta Vallejo‐Yagüe, Renata Vidonscky Lüthold, Angela Edith Schulthess‐Lisibach, Christof Bieri, Michaela Barbier, Pascal Juillerat, Sven Streit, Jennifer Inauen.

J.I., S.S. and K.T.J. conceptualised the study design. J.I. and S.S. secured the funding for the project, provided resources for the study, and supervised the research. The methodology for this study was developed by J.I., K.R.W., S.C.Z. and Y.M. C.T., Y.M., K.R.W., S.V. and M.Z. carried out the data collection and C.T. administered the project. Formal analysis was conducted by Y.M., C.T., K.R.W., E.F., S.C.Z. and J.I. C.T., Y.M., K.R.W., S.V., K.T.J., R.V.L., E.V.‐Y., A.E.S.‐L., M.Z., C.B. and M.B. developed the intervention tools. The original draft of the manuscript was written by K.R.W., E.F., C.T., Y.M., S.C.Z. and J.I. Writing review and editing were done by K.R.W., E.F., C.T., J.I., Y.M., S.C.Z. and other authors. All authors approve the final version.

## Conflicts of Interest

The authors declare no conflicts of interest.

## Supporting information


**Data S1:** Supporting information.

**Data S2:** Supporting information.

## Data Availability

The intervention materials will be publicly accessible via the University of Bern's data repository BORIS upon completion of the trial: https://doi.org/10.48620/87773. Before that date, the materials are available from the authors upon request. The qualitative data that support the findings of this study are not publicly available due to privacy or ethical restrictions.
